# Overall risk and risk factors for metachronous peritoneal metastasis after colorectal cancer surgery: a nationwide cohort study

**DOI:** 10.1002/bjs5.50247

**Published:** 2020-01-09

**Authors:** S. Ravn, U. Heide‐Jørgensen, C. F. Christiansen, V. J. Verwaal, R. H. Hagemann‐Madsen, L. H. Iversen

**Affiliations:** ^1^ Department of Surgery Aarhus University Hospital Aarhus Denmark; ^2^ Department of Clinical Epidemiology Aarhus University Hospital Aarhus Denmark; ^3^ Department of Pathology Lillebaelt Hospital Vejle Denmark; ^4^ Danish Colorectal Cancer Group Copenhagen Denmark

## Abstract

**Background:**

This study aimed to identify the cumulative incidence and risk factors of metachronous peritoneal metastasis (M‐PM) from colorectal cancer in patients who had intended curative treatment.

**Methods:**

Patients with colorectal cancer were identified using the Danish Colorectal Cancer Group database for 2006–2015. The Danish Pathology Registry and the Danish National Patient Registry were used to identify M‐PM to 2017. Risk factors were estimated by multivariable absolute risk regression, treating death and other cancers as competing risks. Overall risk and risk differences (RDs) were estimated at 1, 3 and 5 years.

**Results:**

In 22 586 patients with colorectal cancer, the overall risk of M‐PM was reported to be 0·9 (95 per cent c.i. 0·8 to 1·0) per cent at 1 year, 1·9 (1·8 to 2·1) per cent at 3 years and 2·2 (2·0 to 2·4) per cent at 5 years. Advanced tumour category ((y)pT4 *versus* (y)pT1) increased the RD of both M‐PM (2·9 (95 per cent c.i. 2·1 to 3·7) at 1 year and 6·0 (4·9 to 7·2) at 3 years) and lymph node involvement ((y)pN2 *versus* (y)pN0) (2·5 (1·8 to 3·2) at year and 4·3 (3·2 to 5·3) at 3 years). No further increase in risk was observed at 5 years. In a subanalysis, tumour‐involved resection margin (R1 *versus* R0) was associated with M‐PM with a RD of 3·9 (1·6 to 6·2) at 1 year and 5·9 (2·6 to 9·3) at 3 years.

**Conclusion:**

The overall risk of M‐PM in patients with colorectal cancer is low, but is increased in advanced T and N status. Follow‐up of at least 3 years after colorectal cancer surgery may be necessary, given the potential curative treatment of early diagnosed M‐PM.

## Graphical abstract

In a register‐based study of 22 586 Danish patients with colorectal cancer who had intended curative surgery, a low incidence of metachronous peritoneal metastasis (M‐PM) was found. Risk factors for M‐PM were advanced T and N status and right‐sided colonic tumour. Tumour‐involved resection margins and extramural venous invasion were also associated with M‐PM.

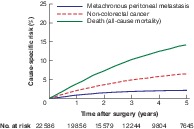

Peritoneal metastases and colorectal tumor

## Abstract (es)

**Antecedentes:**

Este estudio tuvo como objetivo identificar la incidencia acumulada y los factores de riesgo de metástasis peritoneales metacrónicas (*metachronous peritoneal metastases*, M‐PM) del cáncer colorrectal en pacientes que se sometieron al tratamiento curativo previsto.

**Métodos:**

Se identificaron los pacientes con cáncer colorrectal a partir de la base de datos del grupo danés de cáncer colorrectal (*Danish Colorectal Cancer Group*) durante el periodo 2006‐2015. El Registro Danés de Patología (*Danish Pathology Registry*) y el Registro Nacional Danés de Pacientes (*Danish National Patient Registry*) se utilizaron para identificar los casos de M‐PM hasta el 2017. Los factores de riesgo se estimaron mediante una regresión de riesgo absoluto multivariable, tratando la muerte y otros tipos de cáncer como riesgos competitivos. El riesgo general y las diferencias de riesgo (*risk differences*, RD) se estimaron a 1, 3 y 5 años.

**Resultados:**

De los 22.586 pacientes con CCR, el riesgo global de M‐PM fue del 0,9% (i.c. del 95%: 0,8 a 1,0) al año, 1,9 (i.c. del 95%: 1,8 a 2,1) a los 3 años y 2,2 (i.c. del 95%: 2,0 a 2.4) después de 5 años. El estadio T tumoral avanzado ((y) pT4 *versus* (y) pT1) aumentó el riesgo de M‐PM, DR a 1 año: 2,9% (i.c. del 95%: 2,1 a 3,), 3 años: 6,0 (i.c. 95% 4,9 a 7,2), así como la afectación de los ganglios linfáticos ((y) pN2 *versus* (y) pN0), 1 año: 2,5 (i.c. 95% 1,8 a 3,2), 3 años: 4,3 (i.c. 95% 3,2 a 5,3). No se observó un aumento adicional en la DR después de 5 años. Los márgenes de resección tumoral (R1 *versus* R0) se asociaron con una DR a 1 año de 3,9 (i.c. del 95% 1,6 a 6,2), y a 3 años de 5,9 (i.c. del 95% 2,6 a 9,3) de riesgo de M‐PM en un subanálisis.

**Conclusión:**

El riesgo global de M‐PM en el cáncer colorrectal en pacientes es bajo, pero aumenta en las categorías de estadios T y N avanzados. Puede ser necesario un seguimiento de al menos 3 años después de la cirugía de CCR, dado el tratamiento potencialmente curativo de la M‐PM diagnosticada precozmente.

## Introduction

The long‐term survival of patients with colorectal cancer has improved significantly over the past few years; in Denmark, the relative 5‐year survival rate increased from 58–59 per cent in 2001–2004 to 63–65 per cent in 2009–2012^1^. The improvements made so far may be related to several factors, including multidisciplinary team management, the introduction of minimally invasive surgery, implementation of total mesorectal and complete mesocolic excision, specialization and centralization of treatments, pathological/molecular evaluations, and general improvements in radiological assessments, radiotherapy and medical oncology^1^, ^2^. However, recurrence is still an issue.

Registry‐based studies have reported the incidence of metachronous peritoneal metastasis (M‐PM) to be 3·5 per cent at a median of 18 months after diagnosis^3^, rising to 6 per cent within 5 years^4^. Risk factors identified for M‐PM include advanced T and N categories^3^, ^5^, ^6^, ^7^, ^8^, bowel perforation, emergency surgery^5^, ^7^, ^9^ and non‐radical resection^3^, ^5^.

Two different strategies for the prevention and early detection of M‐PM have been proposed, including prophylactic adjuvant hyperthermic intraperitoneal chemotherapy (HIPEC)^10^ and early detection with second‐look surgery plus HIPEC^11^. However, selection of appropriate patients for these treatment options was based on previously identified risk factors for M‐PM^5^, ^12^, and needs further investigation.

This study aimed to describe the overall 5‐year risk of developing M‐PM in patients with colorectal cancer, and to identify risk factors for M‐PM following intended curative surgery.

## Methods

A nationwide registry‐based cohort study was conducted in Denmark according to the STROBE criteria^13^. All 5·8 million Danish citizens have access to a public tax‐supported healthcare system and are assigned a unique ten‐digit personal registration number, enabling unambiguous individual‐level record linkage between registers.

Patients diagnosed with colorectal cancer in the Danish Colorectal Cancer Group database between 2006 and 2015 were identified. In March 2014, the implementation of a national screening programme with faecal immunochemical testing^14^ led to diagnoses of both symptomatic and asymptomatic patients with colorectal cancer. During the study period, national guidelines^15^ recommended that follow‐up of patients with colorectal cancer should include, as a minimum, CT of the thorax and abdomen at 12 and 36 months after surgery.

The Danish Data Protection Agency approved the study (number 1‐16‐02‐441‐16). Ethical approval is not required for registry‐based studies.

**Figure 1:**
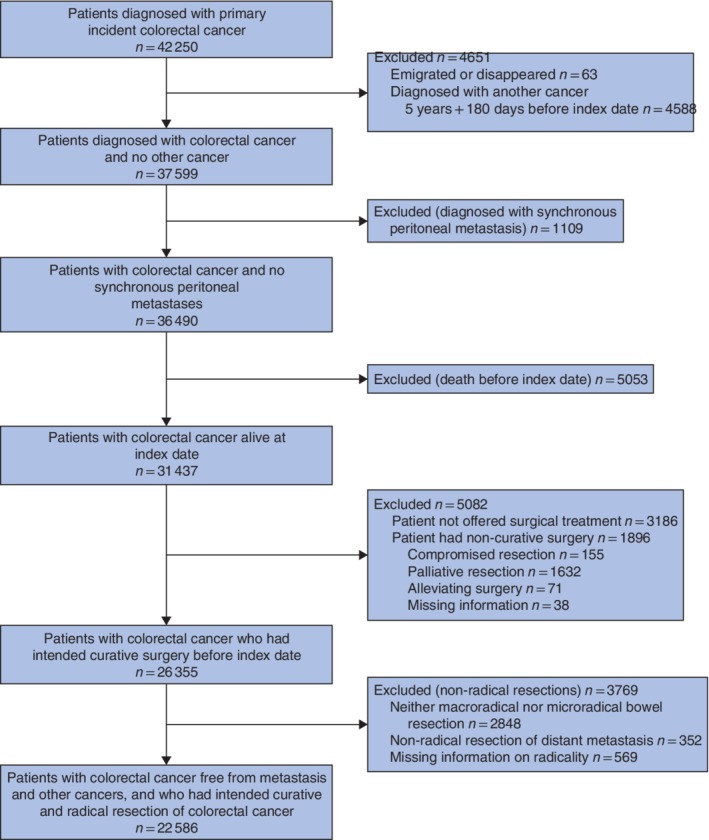
Flow diagram of patients diagnosed with colorectal cancer in 2006–2015 The index date is 180 days after the date of colorectal cancer diagnosis.

Inclusion and exclusion criteria for the study population are summarized in *Fig*. 1. Patients were included if they had undergone a pathologically confirmed R0 or R1 bowel resection for colorectal cancer. Patients with metastasis to liver and/or lungs were included if the surgery was performed with curative intent. The date of colorectal cancer diagnosis plus 180 days was considered as the index date. To reduce immortal time bias, the index date was considered as the beginning of follow‐up^16^, ^17^.

Patients were excluded if they had emigrated or had been diagnosed with other (non‐colorectal cancer) malignancies within a period of 5 years plus 180 days before the index date. Finally, patients were excluded if diagnosed with synchronous peritoneal metastasis (S‐PM) (identified before the index date) or if they had died between the diagnosis and index dates. Synchronous PMs were defined as PMs identified within 180 days of the diagnosis of colorectal cancer^18^, ^19^.

### Registries

Data from the Danish Colorectal Cancer Group database were merged to identify M‐PM and cross‐check follow‐up. In particular, the Danish National Patient Registry provided information about diagnostic coding of PM, the Danish National Pathology Registry was reviewed for histologically proven PM, and the Danish Civil Registration System for follow‐up and vital status. The Danish Colorectal Cancer Group database contains information about all patients with first‐time colorectal cancer since 2001, with data completeness of more than 95 per cent. The database also contains information on patient characteristics, radiological evaluation, surgical and oncological treatment, pathology reporting, and the postoperative course within 30 days of surgery^20^.

The Danish National Patient Registry provides longitudinal data from 1977 regarding administrative and clinical data, and contains information about hospital admissions and outpatient contacts with the healthcare system. Diagnoses were recorded using ICD‐10 codes from 1994, whereas treatment and procedures are registered by using a Danish version of the Nordic Medico‐Statistical Committee (NOMESCO) Classification of Surgical Procedures.

The Danish National Pathology Registry was established in 1997, and all pathological examinations performed in Denmark are registered following a uniform guideline. Each specimen is linked to the personal registration number, the hospital department responsible for treatment, the date of request, the specific Danish Systematized Nomenclature of Medicine codes^21^, and other sources of data.

The Danish Civil Registration System is an administrative register established in 1968 to record information about residency and vital status of all Danish citizens. The register is updated daily and has a high accuracy, allowing for complete long‐term follow‐up^22^.

### Identification of metachronous peritoneal metastasis

In the Danish National Patient Registry, M‐PM was identified by two ICD‐10 codes: ‘metastasis in the retroperitoneal space or in the peritoneum’ (C786) and ‘metastasis to the ovaries’ (C796). In the Danish National Pathology Registry, M‐PM was identified as a specimen/biopsy with a topography code as peritoneum, combined with a specific morphology code representing metastatic spread from the colon or rectum (*Appendix* *S1*, supporting information).

### Potential risk factors

Variables included age (less than 60, 60–75 or more than 75 years), sex, tumour localization (right colon (caecum and ascending colon), right colonic flexure, transverse colon, left colonic flexure, left colon (descending colon and sigmoid), rectum), surgery (elective or emergency), perforation of the tumour as assessed at operation by the surgeon (no; yes, encapsulated (perforation not free in the peritoneal cavity); or yes, free to the peritoneum), pathologically assessed T category ((y)pT0–1, (y)pT2, (y)pT3 or (y)pT4), pathologically assessed N category ((y)pN0, (y)pN1 or (y)pN2), tumour histology (adenocarcinoma or other), extramural venous invasion (EMVI) (available from 2009), radicality of the resection (R0, no macroscopic or microscopic tumour residual left in resection margins; R1, microscopic tumour residual left 1 mm or less from resection margins (included in 2014 owing to the implementation of new strict national guidelines); R2, macroscopic tumour tissue left during resection of the tumour), and systemic chemotherapy (yes or no).

Information on tumour histology was obtained from the Danish National Pathology Registry (*Appendix* *S1*, supporting information). Data on systemic chemotherapy before the index date were obtained from the Danish National Patient Registry using the specific NOMESCO codes for systemic chemotherapy (*Appendix* *S1*, supporting information); however, no information about cycles, doses or frequency was available^23^.

### Statistical analysis

Patient characteristics and demographics are presented as categorical variables by counts and percentages.

Patients were followed up from the index date to the date of diagnosis of M‐PM or non‐colorectal cancer, death or to 25 January 2017. Cumulative incidence (risk) curves for M‐PM were estimated; all‐cause mortality (death) and diagnosis of non‐colorectal cancer were considered to be competing risks^24^, ^25^.

Analysis of potential risk factors was done as a complete‐case analysis: only patients with no missing values for potential risk factors were included. To assess 1‐, 3‐ and 5‐year risk differences (RDs) with 95 per cent c.i. for M‐PM associated with each risk factor, a multivariable absolute risk regression model including all risk factors (except radicality and EMVI) was conducted, adjusting for year of colorectal cancer diagnosis and co‐morbidity, as assessed by the Charlson Co‐morbidity Index (CCI) (categorized as low (score 0), medium (score 1–2) or high (score greater than 2)). Death and non‐colorectal cancer were considered as competing risks^26^, ^27^.

Details of radicality (R1) and EMVI were not available for the whole study period, and were therefore investigated in subgroups of the cohort restricted to relevant calendar periods, using models adjusted only for age, sex and co‐morbidity (CCI) owing to the small number of patients with M‐PM.

Statistical analysis was performed using STATA® software release IC15 (StataCorp, College Station, Texas, USA).

## Results

Overall, 42 250 patients with colorectal cancer were identified in the DCCG database, 22 586 of whom met the study criteria (*Fig*. 1). These patients did not have a cancer diagnosis 5 years before the diagnosis of colorectal cancer, were assessed negative for synchronous PMs, and underwent intended curative resection (R0 or R1) of the tumour, including concomitant procedures if liver and/or lung metastases were present. Patient, tumour and treatment characteristics at the time of colorectal cancer diagnosis are shown in *Table* 1.

**Table 1:** Baseline characteristics of patients with colorectal cancer, diagnosed in 2006–2015, undergoing intended curative and macroscopically radical surgery for the primary colorectal tumour

	No. of patients (*n* = 22 586)
**Age at diagnosis of colorectal cancer (years)**	
< 60	4034 (17·9)
60–75	11 069 (49·0)
> 75	7483 (33·1)
**Sex**	
F	10 548 (46·7)
M	12 038 (53·3)
**Charlson Co‐morbidity Index score**	
0	13 289 (58·8)
1–2	3991 (17·7)
> 2	5306 (23·5)
**Tumour localization**	
Right colon	4914 (21·8)
Right colonic flexure	968 (4·3)
Transverse colon	1110 (4·9)
Left colonic flexure	607 (2·7)
Left colon	7260 (32·1)
Rectum	7724 (34·2)
Colon unspecified	3 (0·0)
**Metastasis to liver or lung at diagnosis of colorectal cancer**	
Yes	91 (0·4)
No	22 495 (99·6)
**Localization of metastasis**	*n* = 91
Liver only	79 (87)
Lung only	7 (8)
Liver and lung	5 (5)
**Priority of surgery**	
Elective	21 261 (94·1)
Emergency*	1322 (5·9)
Missing	3 (0·0)
**Intended operative approach**	
Laparoscopy	12 528 (55·5)
Laparotomy	8569 (37·9)
Robot‐assisted	660 (2·9)
Other minimally invasive†	84 (0·4)
Endoscopy	745 (3·3)
**Tumour perforation**	
No	21 951 (97·2)
Yes, encapsulated	381 (1·7)
Yes, free to peritoneum	254 (1·1)
**(y)pT category** ‡	
T0–T1	2598 (11·5)
T2	3867 (17·1)
T3	13 376 (59·2)
T4	2576 (11·4)
Tx	153 (0·7)
Missing	16 (0·1)
**(y)pN category** §	
N0	14 337 (63·5)
N1	4748 (21·0)
N2	2609 (11·6)
Nx	892 (3·9)
**Microradical surgery**	
Calendar years 2006–2013	*n* = 16 365
Yes, R0¶	16 365 (100)
Calendar years 2014–2016	*n* = 6221
Yes, R0¶	5801 (93·2)
No, R1#	420 (6·8)
**Tumour histology**	
Adenocarcinoma	21 200 (93·9)
Other**	1386 (6·1)
**Extramural venous invasion** ††	
No	10 804 (47·8)
Yes	2862 (12·7)
Missing	1921 (8·5)
n.a.	6999 (31·0)
**Postoperative oncological treatment within 180 days of diagnosis of colorectal cancer**
Yes	5924 (26·2)
No	16 662 (73·8)
**Year of diagnosis of colorectal cancer**	
2006–2007	4123 (18·3)
2008–2009	3945 (17·5)
2010–2011	3993 (17·7)
2012–2013	4304 (19·1)
2014–2015	6221 (27·5)

Values in parentheses are percentages.

* Reason for emergency surgery: ileus (42·0 per cent), perforation (12·8 per cent), other (5·6 per cent), bleeding (0·6 per cent), missing (39·0 per cent).

† Includes (amongst others) transanal total mesorectal excision.

‡ ypT0–1, 298; ypT2, 386; ypT3, 825; ypT4, 149; ypTx, one.

§ ypN0, 1147; ypN1, 347; ypN2, 158; ypNx, seven.

¶ R0, neither macroscopic nor microscopic tumour residual left in resection margins.

# R1 included only from 2014 owing to implementation of new strict national guidelines recommending use and coding of the term ‘not microscopically radical resection’ included microscopic tumour residual left 1 mm or less from resection margins.

** Includes: mucinous adenocarcinoma, low differentiated adenocarcinoma, signet ring cell carcinoma, medullary carcinoma, undifferentiated adenocarcinoma, serrated adenocarcinoma and carcinoma.

†† Data available from 2009. n.a., Not applicable.

### Overall risk of metachronous peritoneal metastases

**Figure 2:**
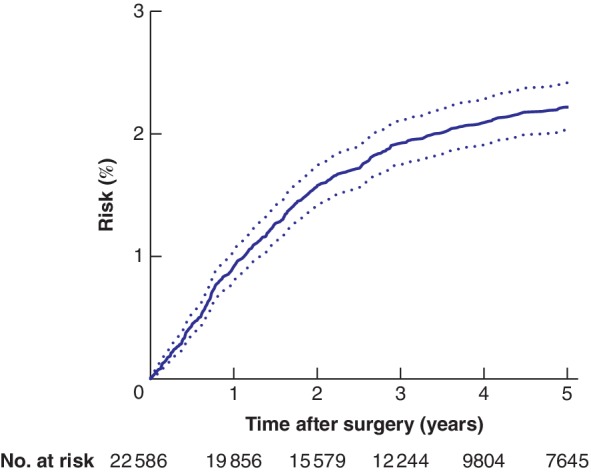
Risk (cumulative incidence) of metachronous peritoneal metastases in Danish patients undergoing intended curative surgery for colorectal cancer in 2006–2015 Dotted lines indicate 95 per cent confidence intervals of risk.

**Figure 3:**
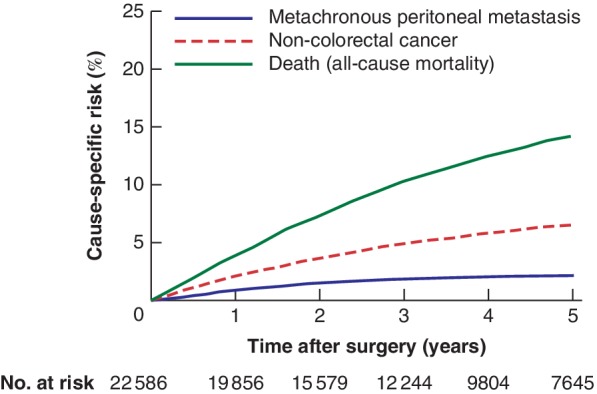
Risk (cumulative incidence) of metachronous peritoneal metastasis, death (all‐cause mortality) and non‐colorectal cancer in Danish patients undergoing intended curative surgery for colorectal cancer in 2006–2015

Some 533 of the 22 586 patients (2·4 per cent) developed peritoneal metastases, among whom 84·4 per cent were identified in the Danish National Patient Registry and 6·0 per cent in the Danish National Pathology Registry; an additional 9·6 per cent were identified in both registries. The overall risk of M‐PM after intended curative surgery for colorectal cancer was 0·9 (95 per cent c.i. 0·8 to 1·0) per cent at 1 year, 1·9 (1·8 to 2·1) per cent at 3 years, and 2·2 (2·0 to 2·4) per cent at 5 years (*Fig*. 2). Death and non‐colorectal cancer were assessed as the major competing risks (*Fig*. 3).

### Risk factors for metachronous peritoneal metastases

Results of the absolute risk regression analyses are shown in *Table* 2. A total of 21 581 patients (95·6 per cent) had complete data for risk factors. The multivariable analysis showed that (y)pT4 status increased the absolute risk by 2·9 (95 per cent c.i. 2·1 to 3·7) per cent at 1 year and by 6·0 (4·9 to 7·2) per cent at 3 years. Compared with a (y)pN0 tumour, (y)pN2 status was associated with a 2·5 (1·8 to 3·2) per cent risk of M‐PM at 1 year and a 4·3 (3·2 to 5·3) per cent risk at 3 years. Estimates of the 5‐year RD showed similar associations to the 3‐year estimates (data not shown).

**Table 2:** Multivariable absolute risk differences for metachronous peritoneal metastases 1 and 3 years after intended curative colorectal cancer surgery

	Multivariable adjusted absolute risk difference (%)*	
	1 year†	3 years†
**Age at diagnosis of colorectal cancer (years)**		
< 60	0 (reference)	0 (reference)
60–75	−0·2 (−0·6, 0·2)	−0·5 (−1·1, 0·2)
> 75	−0·5 (−0·9, 0·0)	−1·0 (−1·7, −0·4)
**Sex**		
F	0 (reference)	0 (reference)
M	0·1 (−0·2, 0·4)	0·3 (−0·1, 0·7)
**Tumour localization**		
Left colon	0 (reference)	0 (reference)
Left colonic flexure	0·6 (−0·5, 1·6)	0·6 (−0·9, 2·2)
Transverse colon	0·2 (−0·5, 0·9)	0·2 (−0·9, 1·3)
Right colonic flexure	0·1 (−0·6, 0·7)	−0·2 (−1·2, 0·9)
Right colon	0·5 (0·1, 0·9)	0·6 (0·0, 1·3)
Rectum	−0·1 (−0·4, 0·2)	−0·3 (−0·8, 0·1)
**Priority of surgery**		
Elective	0 (reference)	0 (reference)
Emergency	0·9 (−0·1, 1·9)	1·9 (0·5, 3·4)
**Tumour perforation**		
No	0 (reference)	0 (reference)
Yes, encapsulated	−1·0 (−2·1, 0·1)	−0·3 (−2·5, 1·9)
Yes, free to peritoneum	−0·1 (−2·2, 2·0)	−0·2 (−3·4, 3·1)
(**y)pT category**		
T1	0 (reference)	0 (reference)
T2	−0·1 (−0·3, 0·2)	0·0 (−0·4, 0·4)
T3	0·1 (−0·2, 0·3)	0·6 (0·2, 1·0)
T4	2·9 (2·1, 3·7)	6·0 (4·9, 7·2)
**(y)pN category**		
N0	0 (reference)	0 (reference)
N1	0·5 (0·1, 0·9)	1·3 (0·7, 2·0)
N2	2·5 (1·8, 3·2)	4·3 (3·2, 5·3)
**Tumour histology**		
Adenocarcinoma	0 (reference)	0 (reference)
Other	0·2 (−0·6, 0·9)	0·4 (−0·8, 1·5)
**Postoperative chemotherapy within 180 days of colorectal cancer diagnosis**		
No	0 (reference)	0 (reference)
Yes	0·0 (−0·4, 0·4)	−0·2 (−0·8, 0·5)
**Extramural venous invasion** ‡		
No	0 (reference)	0 (reference)
Yes	2·3 (1·7, 3·0)	3·4 (2·5, 4·4)
**Radicality of surgery** §		
R0	0 (reference)	0 (reference)
R1	3·9 (1·5, 6·2)	5·9 (2·6, 9·3)

Death and other cancer were treated as competing risks. Values in parentheses are 95 per cent confidence intervals.

* A total of 21 581 complete cases were included in the multivariable analysis, adjusted for all risk factors in the table, including year of diagnosis and co‐morbidity (Charlson Co‐morbidity Index score).

† The baseline risk of metachronous peritoneal metastases for a reference person was 0·2 (95 per cent c.i. 0 to 0·7) per cent at 1 year and 0·6 (0 to 1·5) per cent at 3 years.

‡ Data available from 2009, adjusted only for age, sex and co‐morbidity, for a restricted group of 13 222 patients (complete cases in the multivariable analysis and complete information for extravenous mural invasion).

§ Data available from 2014, adjusted only for age, sex and co‐morbidity, for a restricted group of 5861 patients (complete cases in the multivariable analysis and complete information for R1 resection available from 2014 and 2015).

In addition, right‐sided colonic cancers demonstrated an absolute risk of 0·5 (95 per cent c.i. 0·1 to 0·9) per cent at 1 year and 0·6 (0·0 to 1·3) per cent at 3 years, compared with left‐sided colonic cancers. Emergency surgery increased the risk by 0·9 (−0·1 to 1·9) per cent at 1 year and 1·9 (0·5 to 3·4) per cent at 3 years. All estimates of the 5‐year RD showed similar associations to the 3‐year estimates (data not shown).

EMVI was associated with an absolute risk of 2·3 (95 per cent c.i. 1·7 to 3·0) and 3·4 (2·5 to 4·4) per cent at 1 and 3 years respectively, whereas the corresponding absolute RD for microscopic tumour‐involved resection margins (R1) was 3·9 (1·5 to 6·2) and 5·9 (2·6 to 9·3) per cent.

In the multivariable absolute risk regression analysis, tumour perforation did not correlate with an increased risk of M‐PM. Therefore, a *post hoc* analysis was conducted to compare mortality in patients with tumour perforation and in those without. This analysis showed that the risk of death was substantially higher in patients with tumour perforation (data not shown); thus the null result could be due to competing events.

The baseline risk of M‐PM for a reference person (the risk in someone who presented with the reference value for all co‐variables) was 0·6 (95 per cent c.i. 0·0 to 1·5) per cent at 3 years. *Table* 2 shows the absolute RD for each factor; this should be added to the baseline risk to obtain the predictive risk of M‐PM for a specific patient. According to this analysis, a patient with (y)pT3 N1 rectal cancer undergoing elective surgery would have an estimated total risk of M‐PM of 2·2 per cent after 3 years: 0·6 per cent (overall risk) −0·3 per cent (rectal cancer) + 0·6 per cent ((y)pT3) + 1·3 per cent ((y)pN1) + 0 per cent (elective surgery).

In contrast, a patient with a right‐sided (y)pT4 N2 colonic tumour undergoing emergency surgery would have an estimated risk of M‐PM of 13·4 per cent (0·6 per cent (overall risk) + 0·6 per cent (right colonic cancer) + 6 per cent ((y)pT4) + 4·3 per cent ((y)pN2) + 1·9 per cent (emergency surgery)) at 3 years after intended curative surgery. As EMVI and radicality were not multivariably adjusted, the estimated RDs associated with these variables should be interpreted with caution.

## Discussion

In this large population‐based registry study, the risk of M‐PM was nearly 1 per cent after 1 year, increasing to 2·2 per cent within 5 years. Overall, (y)pT4 and (y)pN2 categories were assessed as independent risk factors for M‐PM, driving the increased risk between 1 and 3 years. All estimates of the 5‐year RD showed similar associations to the 3‐year estimates. Additionally, right‐sided colonic cancers and tumours that required emergency surgery independently increased the risk of M‐PM. EMVI and microscopic tumour‐involved margins (R1 resections) were also associated with an increased risk, although the estimated RDs for these may require further analysis.

In addition, the present study excluded patients who had non‐colorectal cancer within 5 years before the colorectal cancer diagnosis, and non‐colorectal cancer diagnosed during follow‐up (*Fig*. 3) was considered as a competing risk to minimize the chances of including PMs that originated from other locations.

Previous studies^26^, ^27^ have reported different ranges for M‐PM, a variation that may be explained by methodological issues and different time periods. In a prospective clinical study^6^, 5·3 per cent (135 of 2542) of the patients were diagnosed with M‐PM by CT. All patients included were diagnosed with colorectal cancer between 1989 and 1999, and the incidence of M‐PM was not reported at specific time points^6^. In other clinical studies, rates of up to 19 per cent were reported, although these studies analysed M‐PM before the further optimization of colorectal surgery^26^. In comparison, registry‐based studies^3^, ^5^ have found the risk of M‐PM to be in accordance with the results reported here.

In the present study, strict inclusion criteria were used, which could explain the lower incidence compared with that reported in other studies. Other reports included patients receiving a R2 resection, distinguished between synchronous and metachronous PMs as early as 30 days after colorectal cancer resection, included patients alive at 30 days after surgery, and did not report any information regarding the presence of other cancers. However, the low incidence observed in the present study may be related to the multidisciplinary improvement in surgical, radiological, oncological and pathological management of colorectal cancer.

The potential risk factors for M‐PM were in accordance with those of previous studies^5^, ^28^, including T and N categories, surgical radicality and emergency surgery as independent risk factors for M‐PM. Several other studies^3^, ^8^, ^12^, ^29^ have reported similar associations. Still, the identification of patients at high risk of developing M‐PM with the aim of including them in preventive and prophylactic clinical trials is challenging^30^. The effects of early detection with second‐look surgery including HIPEC were investigated in 41 patients with colorectal cancer 1 year after curative resection with no signs of clinical, biochemical or radiological signs of recurrence^11^. The study documented PMs in 23 of the 41 patients after the second‐look procedure; these metastases were treated with cytoreductive surgery and HIPEC, whereas other patients were treated using HIPEC alone. The results suggested a beneficial overall survival and low recurrence rate of PM at a median follow‐up of 30 (9–109) months^11^. However, the patients selected for that study included those with S‐PM, synchronous ovarian metastasis and tumour perforation. In this respect, the results of the present study suggest that patients with tumour perforation represent a very fragile subgroup with high short‐term mortality. This should be taken into consideration when including these patients in future trials.

Of note, the impact of cytoreductive surgery and HIPEC in the present cohort was not investigated as it was considered beyond the scope of this analysis, which aimed to identify risk factors for M‐PM. The recently published RCT^31^ investigating adjuvant HIPEC in patients with T4 tumours or perforated colorectal cancer (COLOPEC trial), documented no benefit of adjuvant HIPEC in terms of peritoneal metastases‐free survival at 18 months. However, during follow‐up, PMs were reported in 21 per cent of the overall study population, indicating the magnitude of the risk in patients with high‐risk colorectal cancer^31^.

Given the potential for curative treatment of M‐PM, the present results indicate that follow‐up of at least 3 years after colorectal cancer surgery may be warranted to detect the majority of incident cases.

Although the registries provide complete information regarding follow‐up, allowing assessment of the risk of M‐PM at specific time points after colorectal cancer surgery, a general limitation of using these registry‐based data is that the assessment of PM may not be uniform; the registration originates from diverse centres throughout Denmark and, according to the longitudinal design, treatments changed over the years^32^. This might introduce information bias, although data were adjusted for the year of colorectal cancer diagnosis in the multivariable absolute risk regression model.

In addition, M‐PM was identified by the use of two nationwide registries: the Danish National Pathology Registry, where the diagnosis of M‐PM is based on pathological examination of the tissue specimen, and/or the Danish National Patient Registry, where the diagnosis is based on the clinician's reporting of an ICD‐10 diagnosis. Thus, the registration of PMs may be reported insufficiently.

Finally, the statistical model applied in the present study does not restrict probabilities to the interval of 0–1. The c.i. of some baseline risk estimates included negative numbers, in which case the lower limit was set to zero. Furthermore, the prediction model presented here has not been validated. The aim of the study was to determine individual risk factors rather than to predict M‐PM, and thus the model should not be used for prediction in future patients without external validation.

## Supporting information


**Appendix S1.** Codes from the Nordic Medico‐Statistical Committee Classification of Surgical Procedures identifying ‘administered systemic chemotherapy’ in the Danish National Patient Registry, and codes from the Danish Systematized Nomenclature of Medicine identifying ‘tumour histology’ and ‘peritoneal metastases’ in the Danish National Pathology Registry
