# Supplementary material for: Overall risk and risk factors for metachronous peritoneal metastasis after colorectal cancer surgery: a nationwide cohort study
Source: BJS Open. 2020 Jan 9;4(2):284–92. doi: 10.1002/bjs5.50247 (PMC7093782; doi:10.1002/bjs5.50247)
Supplement: Supplementary file 1 — Appendix S1. Codes from the Nordic Medico‐Statistical Committee Classification of Surgical Procedures identifying ‘administered systemic chemotherapy’ in the Danish National Patient Registry, and codes from the Danish Systematized Nomenclature of Medicine identifying ‘tumour histology’ and ‘peritoneal metastases’ in the Danish National Pathology Registry [file BJS5-4-284-s001.docx]

**BJS5_50247**

**Overall risk and risk factors for metachronous peritoneal metastasis after colorectal cancer surgery: a nationwide cohort study**

**S. Ravn, U. Heide-Jørgensen, C. F. Christiansen, V. J. Verwaal, R. H. Hagemann-Madsen and L. H. Iversen**

## **Appendix S1** Codes from the Nordic Medico-Statistical Committee Classification of Surgical Procedures identifying ‘administered systemic chemotherapy’ in the Danish National Patient Registry, and codes from the Danish Systematized Nomenclature of Medicine identifying ‘tumour histology’ and ‘peritoneal metastases’ in the Danish National Pathology Registry

| **Administered systemic chemotherapy (Yes, no), identified in the Danish National Patient Registry:** |  |
| --- | --- |
| BWHA1 | Basic cytostatic treatment |
| BWHA108 | Oxaliplatin |
| BWHA110 | 5-fluorouracil |
| BWHA2 | Complex cytostatic treatment |
| BWHA212 | Irinotecan |
| BWHA222 | Capecitabin+oxaliplatin |
| BWHA231 | 5-fluorouracil+oxaliplatin |
| BWHA233 | 5-fluorouracil+irinotecan |

| **Tumour histology (adenocarcinoma, other), identified in the Danish National Pathology Registry:** | T (Localization) | M (tumour type) |
| --- | --- | --- |
| Adenocarcinoma | T67000  T67005  T67100  T67200  T67300  T67400  T67500  T67600  T67700  T68000  T68005  T67920  T67921  T68910  T67965  T67966  T67995  T67996  T65902  T65900  T67010  T67105  T67210  T67310  T67410  T67510  T67610  T67710  T67925  T68010 | M81403  M82103 |
| Low differentiated adenocarcinoma |  | M82313 |
| Medullary carcinoma |  | M85103 |
| Undifferentiated adenocarcinoma |  | M80203 |
| Mucinous adenocarcinoma |  | M84803 |
| Signet ring cell carcinoma |  | M84903 |
| Carcinoma |  | M80103 |
| Serrated adenocarcinoma |  | M82133 |

| **Peritoneal metastases, identified in the Danish National Pathology Registry by a combination of a T(localization)-code, morphology code with the origin in the colon/rectum.** | | |
| --- | --- | --- |
| Localization | Morphology | Origin |
| TY4400 (Peritoneum)  TY4410 (Peritonealt mesotel)  T64120 (serosa, small bowel)  T67090 (serosa, colon)  T68065 (serosa, rectum)  T74050 (serosa, urinary bladder)  T82500 (serosa, uterus)  T66020 (serosa, appendix)  T6X942 (cytology, peritoneal fluid)  T6X944 (cytology, peritoneal scrape)  T6x940 (cytology, peritoneum)  T63850 (omentum)  T63860 (greater omentum)  T63870 (lesser omentum)  T44300 (abdominal wall)  TY4220 (umbilicus)  TY4230 (umbilical region)  T18010 (ligament)  T18030 (hepatic falciform ligament)  T86300 (ligamentum latum)  T8630A (ligamentum latum, right)  T8630B (ligamenttum latum, left)  T86700 (ligamentum rotundum)  T8670A (ligamentum rotundum, right)  T8670B (ligamentum rotundum, left) | M81403 (adenokarcinom)  M81404  M81406  M81407  M81409  M84803 (mucinous adenocarcinoma)  M84804  M84806  M84807  M84809  M84903 (signet ring cell carcinoma)  M84904  M84906  M84907  M84809  M80103 (carcinoma)  M80104  M80106  M80107  M80109  M82313 (low differentiated adenocarcinoma)  M82314  M82316  M82317  M82319  M85743 (adenocarcinoma with neuroendocrine differentiation)  M85744  M85746  M85747  M85749  M814F3 (adenocarcinoma on the basis of immune profile)  M814F4  M814F6  M814F7  M814F9  M85103 (medullary carcinoma)  M85104  M85106  M85107  M85109 | ÆF4450 colon/rectum |
